# Elevated CO_2_ Altered Rice VOCs Aggravate Population Occurrence of Brown Planthoppers by Improving Host Selection Ability

**DOI:** 10.3390/biology11060882

**Published:** 2022-06-08

**Authors:** Yanhui Wang, Runzhao Li, Xiaohui Wang, Xiaowei Liu, Fajun Chen

**Affiliations:** Department of Entomology, College of Plant Protection, Nanjing Agricultural University, Nanjing 210095, China; 2018102061@njau.edu.cn (Y.W.); 2020102066@stu.njau.edu.cn (R.L.); huihuisfs@163.com (X.W.); 2019202049@njau.edu.cn (X.L.)

**Keywords:** elevated CO_2_, rice plants, volatile organic compounds, *Nilaparvata lugens*, host selection behavior

## Abstract

**Simple Summary:**

In recent years, the atmospheric CO_2_ concentration was increasing continuously, which has led to the change in the photosynthesis and chemical composition of rice plants. The growth and development of brown planthopper (BPH) *Nilaparvata lugens* are further affected. Plants release volatile organic compounds (VOCs) to mediate intra- and inter-specific interactions with other organisms in the surrounding environment. Therefore, here we aim to explore the effect of rice VOCs on the host selection ability of BPH under elevated CO_2_. Among the identified thirty-six rice VOCs, the contents of heptadecane, linalool and limonene from rice plants were significantly decreased under elevated CO_2_. Moreover, we found that the VOCs of rice damaged by BPH were also changed. Undecane, hexadecane, nonanal and 2,6-diphenylphenol from BPH-damaged rice plants under elevated CO_2_ were all significantly higher than those from healthy rice plants, which might lead to enhancement of the host selection ability of BPH, eventually aggravating the damage caused by BPH. However, the role of these VOCs in host selection ability of BPH is not clear, and more experiments are needed to verify their function.

**Abstract:**

It is predicted that plant volatile organic compounds (VOCs) are affected by the atmospheric CO_2_ levels rising globally, which further affects the interaction between plants and herbivorous insects, especially the host selection behavior of herbivorous insects. In this study, the effects of elevated CO_2_ on the host-selection behavior of the brown planthopper (BPH) *Nilaparvata lugens*, and the emission of VOCs from the healthy and BPH-damaged rice plants were studied simultaneously to make clear the population occurrence of BPH under global climate change. Compared with ambient CO_2_, elevated CO_2_ significantly increased the host selection percent of BPH for the healthy (CK) and BPH-damaged rice plants, and the host selection percent of BPH for the BPH-damaged rice plants was significantly higher than that for the healthy rice plants under elevated CO_2_, which might be regulated by the transcription levels of *OBP1*, *OBP2* and *CSP8* in BPH due to the upregulated transcriptional levels of these three genes of BPH under elevated CO_2_. In addition, we analyzed and quantified the emission of VOCs in rice plants grown under ambient CO_2_ and elevated CO_2_ by GS-MS. A total of 36 VOCs from rice plants were identified into eight categories, including alkanes, alkenes, alcohols, aldehydes, ketones, esters, phenols and aromatic hydrocarbons. Elevated CO_2_ significantly decreased the contents of heptadecane, linalool and limonene from rice plants compared with ambient CO_2_. Besides, the contents of linalool, phytol, decanal, 1-methyldecalin and 2,6-diphenylphenol from BPH-damaged rice plants under ambient CO_2_, and undecane, hexadecane, nonanal and 2,6-diphenylphenol from BPH-damaged rice plants under elevated CO_2_ were all significantly higher than those from healthy rice plants. The percentage composition of phenols was positively correlated with the host selection rate of BPH. Our study indicates that elevated CO_2_ is beneficial to promote the host selection ability of BPH for rice plants damaged by BPHs due to the changed plant VOCs.

## 1. Introduction

In recent years, the main greenhouse gas CO_2_ concentration has gradually increased. According to the report published by the National Oceanic and Atmospheric Administration (NOAA), the atmospheric CO_2_ concentration increased by nearly 10 ppm in the past five years, and it was still growing continuously [1], and was predicted to reach 800 ppm by the end of the 21st century [2]. As a raw material for photosynthesis, elevated CO_2_ can directly affect the photosynthetic rate of plants [3], further affect the C/N ratio of plant tissues [4,5,6], and simultaneously change the allocation of primary and secondary metabolites in plant tissues [7,8,9,10,11].

Plant secondary metabolites play an essential role in plant adversity adaptation (including insect pests’ damage, etc.). The increase in atmospheric CO_2_ concentration affects the distribution of photosynthetic products by affecting plant photosynthesis, which might lead to changes in the contents of plant secondary metabolites (including terpenes, flavonoids, alkaloids, etc.) and affect plant resistance to insects [12,13]. It was found that the N-based secondary metabolites (e.g., terpenoids) decreased, while the C-based secondary metabolites increased (e.g., total phenols, condensed tannins and flavonoids) under elevated CO_2_ [14,15,16]. Those volatile organic compounds (VOCs) released by plants mainly included alkanes, alkenes, terpenoids, aromatic compounds, etc. [17,18], which were also included in the changes in the composition and contents of plant secondary metabolites caused by elevated CO_2_ [19,20]. The effects of elevated CO_2_ on VOCs differed specifically among plant species and different plant tissues [21]. For example, the isoprene emission from the green tissues of plants decreased with the increase in CO_2_ concentration, while the isoprene emission from woody tissues was not affected by CO_2_ concentration [22,23]. However, the emission of the three most abundantly emitted monoterpenes (α-pinene, sabinene and β-pinene) was inhibited under elevated CO_2_ [24]. Ballhorn et al. [25] also reported that the total release of VOCs from lima bean plants significantly increased in response to elevated CO_2_. In addition, the change of VOCs released by host plants under elevated CO_2_ would affect the interaction between plants and insect herbivores [26,27], indicating potential functions of agricultural and natural ecosystems [28].

Plants release VOCs to mediate intra- and inter-specific interactions with other organisms in the surrounding environment. Herbivorous insects could distinguish host plants releasing VOCs using their olfactory senses in the environment [29,30]. The olfactory receptors of insects are located on the antenna and maxillary palpi. Both organs have olfactory receptor neurons (ORNs), which are covered with different types of olfactory receptors [31,32,33]. Herbivorous insects use the receptors on the antennae and chin whiskers to receive odor molecules. The odor-binding proteins combine with the odor molecules and transport them to the olfactory receptors, thus transmitting tactile information to the brain [34]. The odorant-binding proteins (OBPs), chemosensory proteins (CSPs), odorant degrading enzymes (ODEs) and sensory neuron membrane proteins (SNMPs) play important roles in the insects’ initial identification of host plants, and the OBPs and CSPs are the primary peripheral olfactory proteins that play critical roles in odor detection [35]. Studies have shown that the OBPs of the brown planthopper, *Nilaparvata lugens,* could recognize terpenes and ketones [36,37], and several CSPs were identified from BPH [38].

The brown planthopper (BPH), *N. lugens*, is one of the most severe and destructive economic insect pests of rice crops in Asia [39]. Long-term misuse of pesticides has led to the high resistance of BPH, which threatens natural enemy insects and destroys ecosystem diversity, causing frequent BPH outbreaks and bringing considerable losses to rice production in China [40,41]. Green prevention and biological control should be vigorously promoted to avoid rampant pests caused by insecticide resistance. There were some cases of using plant VOCs to control pests in agricultural production [42,43]. It will be a potential application of using plant VOCs to control insect pests during climate change in the future. In this study, the host-selection behavior of BPH, BPH for the healthy rice plants and the BPH-damaged rice plants was measured under ambient and elevated CO_2_, as well as the expression levels of OBPs (including *OPB1*, *OBP2* and *OBP3*) and CSPs (including *CSP3*, *CSP8* and *CSP10*) in BPH, and the emission of VOCs from the healthy and BPH-damaged rice plants, in order to make clear how plant VOCs from rice plants affect the host-selection behavior of BPH in responding to elevated CO_2_.

## 2. Materials and Methods

### 2.1. CO_2_ Level and Environmental Condition

Two CO_2_ levels, including elevated CO_2_ (800 ppm; predicted level at the end of this century) [44,45] and ambient CO_2_ (400 ppm; current atmospheric CO_2_ level), were set up in separate artificial climate chambers (ACCs; GDN-400D-4-CO_2_, Ningbo Southeast Instrument CO_2_, Ningbo, China) with 14 h light and 27 °C/10 h dark and 26.5 °C, and 70% relative humidity (RH), and each CO_2_ level had three ACCs. CO_2_ gas was supplied to each ACC of elevated CO_2_ all day, and the CO_2_ level was automatically monitored and adjusted once every 20 min. The automatic-control system for the CO_2_ concentrations and ACCs was detailed in Qian et al. [46,47].

### 2.2. Plant Materials and Insect Stocks

A susceptible rice variety, Taichung Native 1, (TN1, carrying no BPH resistance genes) was grown in artificial climate chambers (ACCs; GDN-400D-4-CO_2_, Ningbo Southeast Instrument CO_2_, Ningbo, China) at Nanjing Agricultural University, Nanjing, Jiangsu Province of China (32°03′ N, 118°84′ E). The TN1 rice seeds were soaked in water for 24 h, then placed on wet gauze to accelerate germination, and then sowed in seeding trays (one seed per hole). The TN1 plants grown for 15 days were transplanted into plastic pots (23 cm diameter and 16 cm height) filled with potting soil (three rice plants per pot), watered with nutrition solution [48] every three days. The potted rice plants in each ACC were randomly changed every week to avoid the position effect of pots. The rice plants grown for 50 days (i.e., tillering stage) were selected for the following experiment.

The BPH adults were collected from the paddy fields of Jiangsu Academy of Agricultural Science, Nanjing, Jiangsu Province of China (32°04′ N, 118°88′ E), and they were reared with TN1 rice seedlings for more than 40 generations in ACCs under ambient CO_2_ in the laboratory. The photoperiod, temperature and RH in these ACCs were the same as above.

### 2.3. Host Selection Assays of N. lugen Adults for the Healthy and BPH-Damaged Rice Plants

In this study, the healthy (CK) and BPH-damaged rice plants were used under ambient and elevated CO_2_, i.e., there were four treatments, including ambient CO_2_ + healthy (CK) rice, ambient CO_2_ + BPH-damaged rice, elevated CO_2_ + healthy (CK) rice, and elevated CO_2_ + BPH-damaged rice. The pots with BPH-damaged rice plants were covered with plastic buckets, and five pairs of newly emerged female and male adults of BPH were released onto each rice plant, and then the openings at both ends of plastic buckets were sealed with gauze to prevent the BPHs from escaping. After the inoculation for 24 h, the BPH-damaged and healthy (CK) rice plants were collected, and the rice roots were cleaned and wrapped with tinfoil for the host selection assays and plant VOCs collection.

The effects of the elevated CO_2_ on the host-selection behavior of BPHs for the healthy (CK) and BPH-damaged rice plants were quantified by using a four-chamber olfactometer (PSM4-150; Nanjing Pusen Instrument Co. Ltd., Nanjing, China). The diagonal ends of the four-chamber olfactometer were set as the treatment areas for the above four treatments. An 8 W fluorescent lamp was placed above the four-arm motherboard and the flow meter was adjusted to deliver a consistent airflow of 200 mL/min to both sides. Thirty BPH adults within three days of new emergence were selected randomly and starved for two hours, and then released to the center of the four-arm motherboard to observe their host-selection behavior. If the sampled BPH adults reached the nesting area of one arm within 20 min, the treatment corresponding to that arm was considered as the choice of the released BPH adults. Those BPH adults that did not reach any nesting area within 20 min after release were considered non-responders (i.e., no choice). Three replicates per experiment were set up. The four-arm olfactometers were rotated horizontally by 90° every time the experiment was repeated to avoid the position influence. In order to avoid biases in the behavioral observations between tests, the air compressor was turned off for 10 min and wiped with anhydrous alcohol after each test. The intake pipe was also exchanged after each test. All of the tests were carried out in a clean, uniform, well-ventilated and relatively closed laboratory. The BPH adults fed on rice plants grown under ambient and elevated CO_2_ were collected for the following gene expression analysis of OBP genes and CSP genes.

### 2.4. RNA Extraction, cDNA Synthesis and qRT-PCR Analysis

Six BPH adults (including three females and three males) were collected from each biological replicate of each treatment (i.e., three biological replicates) for RNA isolation to analyze the gene transcript expression levels of OBP genes (including *OPB1*, *OBP2* and *OBP3*) and CSP genes (including *CSP3*, *CSP8* and *CSP10*). Total RNA was isolated from the whole body of BPH adults by using the TRIzol^®^ reagent (Invitrogen, Carlsbad, CA, USA). The concentration and quality of samples were determined by using the NanoDrop^TM^ spectrophotometer (Thermo Scientific, Waltham, MA, USA) and 1.5% agarose gel electrophoresis. The cDNA synthesis was carried out with 100 ng of total RNA via the PrimeScript^TM^ RT reagent Kit with gDNA Eraser (Takara, Osaka, Japan). Reverse transcriptase reactions were performed in a reaction volume of 20 μL. The qRT-PCR was performed with a 7500 real-time PCR detection system (Applied Biosystems, Foster City, CA, USA) by using 1× SYBR^®^ Premix Ex Taq^TM^ (TaKaRa, Osaka, Japan), 2 μL cDNA products (diluted from 20 μL to 200 μL with RNase-free water) and 0.2 μM primers in a final reaction volume of 20 μL. The specific primers for the genes of *OPB1*, *OBP2* and *OBP3* [37], and *CSP3* [49], *CSP8* [50] and *CSP10* [51], and the reference genes *β*-*Actin* [52] and *ug-Actin* [37] were listed in Table 1. The genes’ expression level was quantified following the 2^−ΔΔCt^ normalization method, respectively [53]. The relative expression level was represented as the fold changes by comparing the samples of ambient CO_2_ and elevated CO_2_ treatments. Three technical replicates were performed for cDNA.

### 2.5. Collection and Identification Assays of the VOCs from the Healthy and BPH-Damaged Rice Plants Grown under Ambient and Elevated CO_2_

Plant volatile organic compounds (VOCs) were collected using the dynamic headspace adsorption method from the healthy (CK) and BPH-damaged rice plants grown under ambient and elevated CO_2_, respectively. The device consists of air pump, flow meter, washing cylinder, drying tower, cylinder and adsorption tubes. The reagent consumables used included N-hexane (chromatographically pure) and Tenax (200 nm), transparent screw mouth sample bottle (4 mL volume), brown thread mouth automatic sample injection bottle (caliber 9 mm) (Shanghai amps experimental technology Co. Ltd.; Shanghai, China) and high purity nitrogen. GC-MS (320-MS; Brook Dalton mass spectrometry Co.; Brook, IL, USA) was performed to analyze the species and concentrations of the volatile samples above (shown in Supplementary Materials). GC was equipped with a HP-5 Agilent capillary column (30 m × 0.32 mm × 0.25 μm), and the injector temperature was set at 250 °C; Helium was used as the carrier gas at an average flow rate of 1 mL/min. The MS method was as follows: ionization mode was set at EI 70 eV; the source and transfer line were maintained at the temperature of 230 °C and 280 °C, respectively; and the scanned area reached 50–550 m/Z. The VOCs from healthy and BPH-damaged rice plants grown under ambient and elevated CO_2_ were identified by comparing mass spectra with those of authenticated samples in the database.

### 2.6. Statistical Analysis

All data were analyzed using SPSS 20.0 software (IBM Corporation, Armonk, NY, USA). All measured index values were shown in mean ± standard errors (SE). Two-way ANOVAs were used to analyze the effects of CO_2_ level (ambient CO_2_ versus elevated CO_2_), BPH-damaged treatment (healthy (CK) versus BPH-damaged rice plants) and their interaction on the host-selection rate of BPH adults, and on the relative percent of VOCs (including eight groups, and each type of VOCs) from the healthy (CK) and BPH-damaged rice plants grown under ambient and elevated CO_2_. One-way ANOVA was used to analyze the effect of CO_2_ level on the transcript expression of OBP genes (*OPB1*, *OBP2* and *OBP*3) and CSP genes (*CSP3*, *CSP8* and *CSP10*) of BPH fed on rice plants grown under ambient and elevated CO_2_, In addition, significant differences between the two CO_2_ levels (ambient CO_2_ versus elevated CO_2_) and between two types of rice plants (i.e., healthy (CK) and BPH-damaged rice plants) were analyzed by the independent-sample *t*-test at *p* < 0.05.

## 3. Results

### 3.1. Effect of Elevated CO_2_ on the Host Selection of BPH for the Healthy and BPH-Damaged Rice Plants

**Figure 1:**
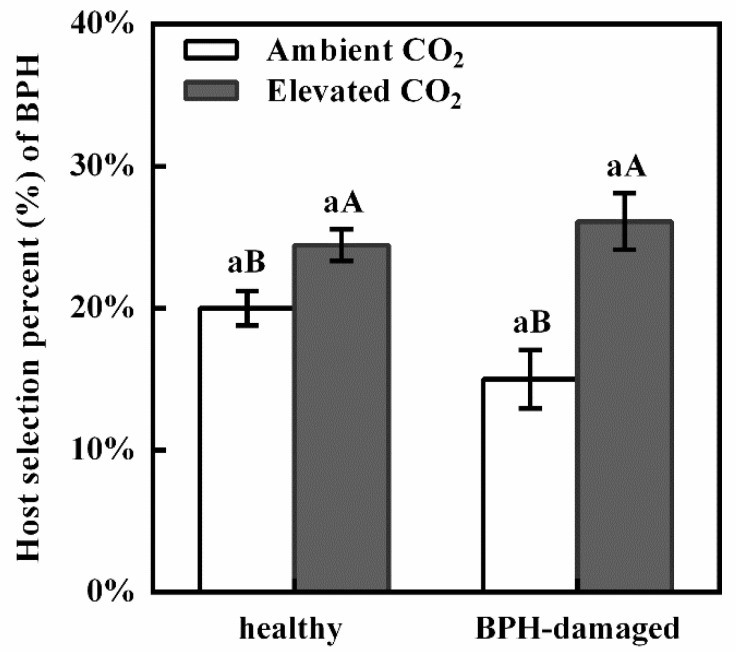
The host selection rate (%) of the adults of brown planthopper (BPH), *Nilaparvata lugens,* for the healthy and BPH-damaged rice plants grown under ambient and elevated CO_2._ (**Note:** Different uppercase and lowercase letters indicate significant differences between ambient and elevated CO_2_ level, and between the healthy (CK) and BPH-damaged rice plants by the independent sample *t*-test at *p* < 0.05, respectively).

Two-way ANOVAs indicated that the CO_2_ level significantly affected the host selection rate of BPH adults (*F* = 22.022, *p* < 0.001), while neither BPH-damaged (*F* = 1.011, *p* = 0.327) nor the interaction between CO_2_ level and BPH-damaged treatment (*F* = 4.045, *p* = 0.058) had a significant effect on the host selection rate of BPH adults (Table 2). The host selection rate of BPHs for the healthy (CK) rice plants was 20.00% under the ambient CO_2_, which was significantly lower than that (24.44%) under the elevated CO_2_ (+22.22%; *p* < 0.05, Figure 1). The host selection rate of BPH for the BPH-damaged rice plants was 15.00% under the ambient CO_2_, which was lower than that (26.11%) under the elevated CO_2_ (+74.07%; *p* < 0.05, Figure 1). Meanwhile there was no significant difference in the host selection rate of BPHs for the healthy (CK) rice plants in contrast to that of the BPHs for the BPH-damaged rice plants, under ambient or elevated CO_2_ (*p* > 0.05, Figure 1).

### 3.2. Relative Expression Levels of OBP and CSP Genes in BPH Adults Fed on Rice Plants Grown under Ambient and Elevated CO_2_

**Figure 2:**
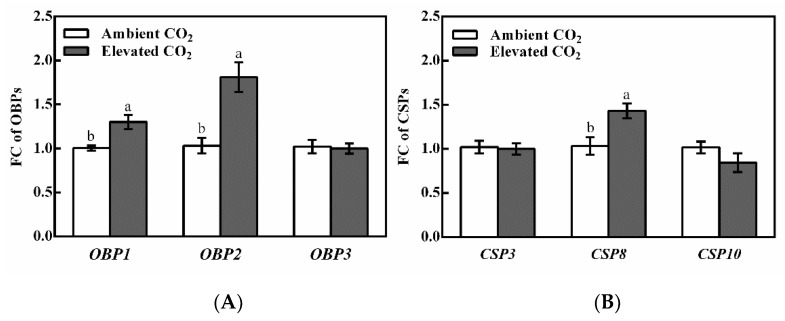
Relative gene expression levels of OBPs (**A**) and CSPs (**B**) in the adults of brown planthopper (BPH), *N. lugens* under ambient and elevated CO_2_. (**Note:** Different lowercase letters indicate significant differences between ambient and elevated CO_2_ level by the independent sample *t*-test at *p* < 0.05).

CO_2_ level significantly affected the relative expression levels of *OBP1* (*F* = 3.471, *p* = 0.006), *OBP2* (*F* = 4.084, *p* < 0.001) and *CSP8* (*F* = 3.076, *p* = 0.007) in BPH adults (Table 2). The relative expression levels of *OBP1* and *OBP2* were significantly upregulated by 29.56% and 75.56% (*p* < 0.05, Figure 2A), as well as the gene expression level of *CSP8*, which was significantly upregulated by 38.56% (*p* < 0.05, Figure 2B) when BPH adults fed on rice plants grown under elevated CO_2_ in contrast to the ambient CO_2_.

### 3.3. Effect of Elevated CO_2_ on the Components and Contents of VOCs from the Healthy and BPH-Damaged Rice Plants

**Figure 3:**
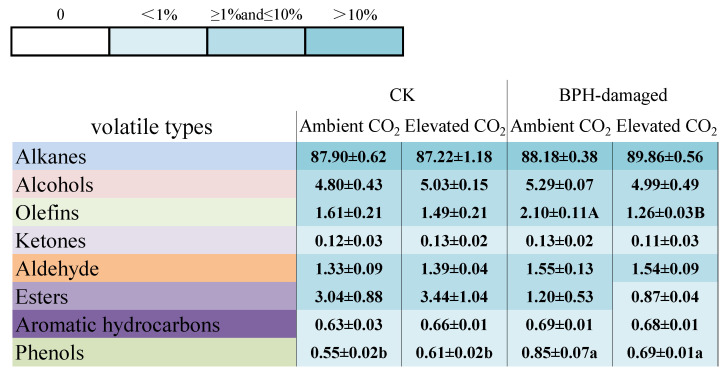
Percentage composition (%) of eight groups of volatile organic compounds (VOCs) from the healthy (CK) and BPH-damaged rice plants grown under ambient and elevated CO_2._ (**Note**: BPH -Brown planthopper, *Nilaparvata lugens*; Different uppercase and lowercase letters indicate significant differences between ambient and elevated CO_2_ level, and between the healthy (CK) and BPH-damaged rice plants by the independent sample *t*-test at *p* < 0.05, respectively. The same is true in the following Figure 4).

Eight chemical groups of VOCs were identified from the healthy (CK) and BPH-damaged rice plants grown under ambient and elevated CO_2_, mainly including alkanes, alcohols, alkenes, ketones, aldehyde, esters, aromatic hydrocarbon and phenols (shown in Figure 3). CO_2_ levels significantly affected the relative percentage composition of alkenes (*F* = 9.195, *p* = 0.016), and BPH-damaged treatment significantly affected the relative percentage compositions of esters (*F* = 9.069, *p* = 0.017), aromatic hydrocarbon (*F* = 6.917, *p* = 0.030) and phenols (*F* = 27.186, *p* < 0.001) from rice plants (Table 2). Moreover, the relative percentage composition of phenols was also significantly affected by the interaction between CO_2_ level and BPH-damaged treatment (*F* = 7.912, *p* = 0.023; Table 2). Compared with ambient CO_2_, elevated CO_2_ had no significant effects on the percentage composition of eight groups of VOCs from the healthy (CK) rice plants (*p* > 0.05), but it significantly reduced the percentage composition of alkenes from the BPH-damaged rice plants (*p* < 0.05, Figure 3). In addition, the percentage composition of phenols from the BPH-damaged rice plants was significantly higher than that from the healthy (CK) rice plants grown under ambient and elevated CO_2_ by 35.29% and 13.11%, respectively (*p* < 0.05, Figure 3).

**Figure 4:**
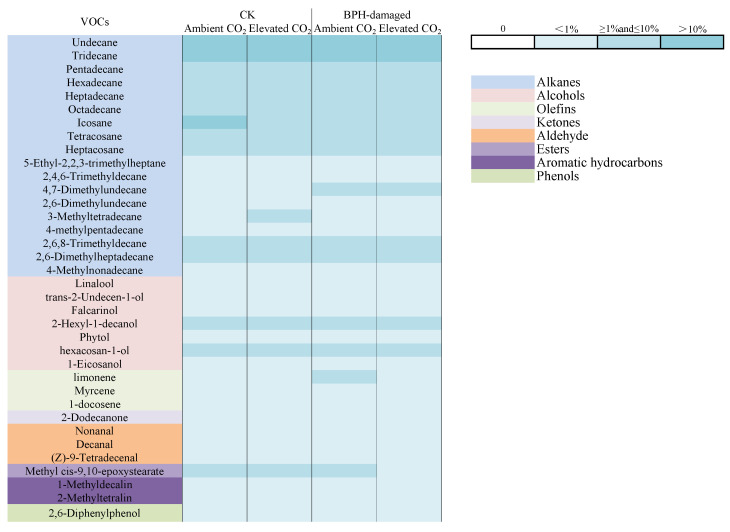
Percentage composition (%) of thirty-six kinds of volatile organic compounds (VOCs) in the healthy (CK) and BPH-damaged rice plants grown under ambient and elevated CO_2__._

Moreover, there were 36 kinds of VOCs identified from the healthy (CK) and BPH-damaged rice plants, the largest components were alkanes with 18 species, the lowest components were phenols with one species (shown in Figure 4). The relative percentages of linalool (*F* = 21.416, *p* = 0.002) and limonene (*F* = 12.893, *p* = 0.007) were significantly affected by the CO_2_ level, and the BPH-damaged treatment significantly affected the relative percentages of hexadecane (*F* = 10.462, *p* = 0.012), linalool (*F* = 6.098, *p* = 0.039), nonanal (*F* = 10.012, *p* = 0.013), methyl cis-9,10-epoxystearate (*F* = 9.180, *p* = 0.016) and 2,6-diphenylphenol (*F* = 27.186, *p* < 0.001) from rice plants (Table 3). The interaction between CO_2_ level and BPH-damaged treatment also significantly affected the relative percentages of heptadecane (*F* = 10.157, *p* = 0.013), linalool (*F* = 9.206, *p* = 0.016), limonene (*F* = 7.430, *p* = 0.026) and 2,6-diphenylphenol (*F* = 7.912, *p* = 0.023) from rice plants (*p* < 0.05, Table 3). Compared with ambient CO_2_, the elevated CO_2_ had no significant effect on the relative percentage contents of the measured 36 kinds of VOCs from the healthy (CK) rice plants (*p* > 0.05), while it significantly decreased the relative percentage contents of heptadecane, linalool and limonene from the BPH-damaged rice plants (*p* < 0.05; Table 4). In addition, the relative percentage components of linalool, phytol, decanal, 1-methyldecalin and 2,6-diphenylphenol from the healthy (CK) rice plants were significantly higher than those from the BPH-damaged rice plants grown under ambient CO_2_ (*p* < 0.05), and the relative percentages of undecane, hexadecane, nonanal and 2,6-diphenylphenol from the BPH-damaged rice plants were significantly higher than those from the healthy (CK) rice plants grown under elevated CO_2_ (*p* < 0.05; Table 4).

### 3.4. Correlation Analysis among the Host-Selection Rate, the Transcript Expression Levels of OBPs and CSPs in BPH Adults and the Relative Percentages of Rice Plant VOCs

**Figure 5:**
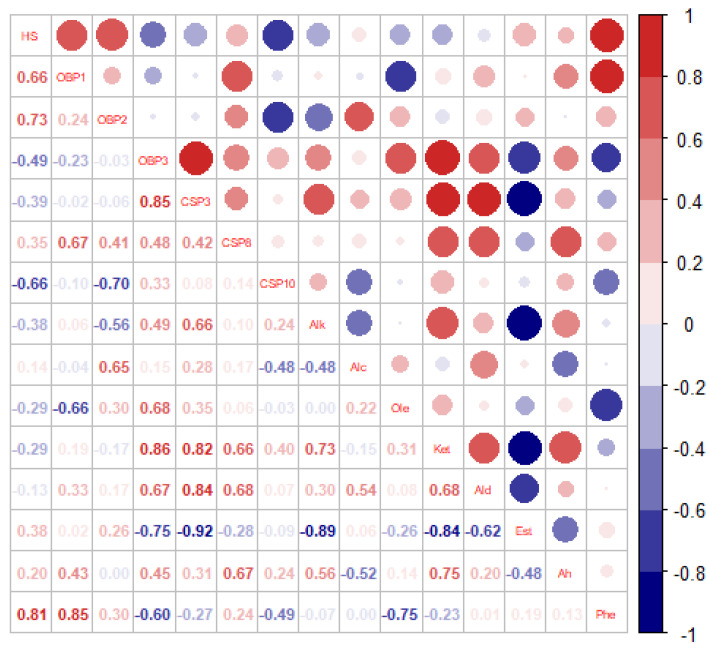
The Pearson analysis of the correlation among the host selection rate, the expression levels of OBPs and CSPs in *N. lugens* adults and the relative percentage compositions of the measured eight groups of volatile organic compounds (VOCs) from rice plants. (**Note:** The scale color of the filled squares indicates the strength of the correlation (r) and whether it is negative (blue) or positive (red). The correlation is stronger when the number corresponding to the color and size of circles is closer to 1 or −1.).

The Pearson analysis showed that the host selection rate of BPH adults was positively correlated with the expression levels of *OBP1* and *OBP2*, and negatively correlated with the expression level of *CSP10* in the BPH adults, and it also indicated that the host selection rate of BPH adults was just positively correlated with the relative percentage composition of phenols (Phe) in the eight chemical groups of VOCs released from rice plants (shown in Figure 5).

## 4. Discussion

It is found that the host selection by phytophagous insects is influenced by many factors, such as the species, quantity and volatile odor of host plants [54,55,56], and environmental factors, including atmospheric CO_2_ concentrations [57,58]. Elevated CO_2_ changes the primary metabolism and secondary metabolism of plants, which affects the host selection of phytophagous insects [57,58]. In this study, we used the plant selection methods through a four-chamber olfactometer to measure the host selection rate of the brown planthopper (BPH), *Nilaparvata lugens* adults for the healthy (CK) and BPH-damaged rice plants. The results indicated that elevated CO_2_ significantly increased the host selection rate of BPH adults for the healthy (CK) and BPH-damaged rice plants compared with the ambient CO_2_. There was no significant difference in the host selection rate of the BPH adults for the healthy (CK) compared to the BPH-damaged rice plants. Interestingly, the host selection of BPH adults for the BPH-damaged rice plants was significantly lower than that for the healthy (CK) rice plants under ambient CO_2_, while it was just the opposite tendency under elevated CO_2_. Qian et al. [58] reported that the western flower thrips, *Frankliniella occidentalis,* have a higher potential host selection ability for the kidney bean, *Phaseolus vulgaris,* damaged by *F*. *occidentalis* under elevated CO_2_. Hu et al. [59] also indicated that BPH favored rice plants damaged by the *Chilo suppressalis*. Since the emission of VOCs from damaged plants could repel or attract herbivorous insects [60,61], it is speculated that elevated CO_2_ would probably aggravate the BPH damage for the BPH-damaged rice plants due to changes in the VOCs released under future climate change.

Herbivorous insects have evolved sensitive olfactory systems, which can sense and deal with specific volatiles emitted by their host plants [62]. It is believed that both OBP and CSP genes carry some functional proteins which participate in the initial recognition of odor perception by capturing hydrophobic odor molecules and transporting them to olfactory receptor neurons through hydrophobic lymph [63,64]. The combination of OBPs and odor molecules is the first biochemical reaction of herbivorous insects’ specificity to identify the external odor substances, which is also the critical component of the first function [65,66]. In this study, the expression levels of *OBP1* and *OBP2* genes were upregulated by elevated CO_2_, which may further enhance the olfactory ability of BPH. Similarly, the expression levels of *OBP2* and *OBP7* in *A. gossypii* adults were also significantly enhanced under elevated CO_2_ compared with the ambient CO_2_ [57]. The expression of OBP genes in insects could be considered a critical factor for their physiological function in regulating insects’ host-selection behavior [67]. In addition, the CSP genes are assumed to have more important functions than OBP genes, and they play an essential role in dissolving and transporting different chemoreceptor fat-affinity ligands, participating in the functional part of olfaction and sensory chemical stimulation [68]. The gene expression level of *CSP8* was upregulated by elevated CO_2_. Consistent results including the upregulated expression of *CSP4* and *CSP6* in *A. gossypii* adults [57], as well as *CSP1* and *CSP1-q* in *F*. *occidentalis,* under elevated CO_2_ compared with ambient CO_2_ [58] were also found. Moreover, the host selection of BPH was positively correlated with the expression levels of OBPs. These results indicated that the transcription levels of OBP genes and CSP genes of BPH upregulated under elevated CO_2_, improving the host selection behavior of BPH adults at the molecular level. On the one hand, the host selection behavior of BPH responses to elevated CO_2_ in the future might aggravate the risk of BPH damage. On the other hand, it might provide a theoretical basis for field pest control under climate change.

The emission of VOCs from plants is affected by individual biotic and abiotic stresses, such as insect feeding, CO_2_ and so on [69]. In this study, thirty-six kinds of VOCs belonging to eight chemical groups were detected from the healthy (CK) and BPH-damaged rice plants, mainly including alkanes, olefins, alcohols, aldehydes, ketones, esters, phenols and aromatic hydrocarbons. Among them, alkanes accounted for more than 87%, which was similar to that detected by Ghaninia and Tabari [70]. We also found that, compared with ambient CO_2_, elevated CO_2_ significantly decreased the relative percentage compositions of heptadecane, linalool and limonene from the BPH-damaged rice plants. The relative percentage composition of phenols from the BPH-damaged rice plants was significantly higher than that from the healthy (CK) rice plants under ambient and elevated CO_2_, respectively. In addition, the relative percentage compositions of linalool, phytol, decanal, 1-methyldecalin and 2,6-diphenylphenol from the BPH-damaged rice plants grown under ambient CO_2_, and the relative percentage compositions of undecane, hexadecane, nonanal and 2,6-diphenylphenol from the BPH-damaged rice plants grown under elevated CO_2_ were significantly higher than those from the healthy (CK) rice plants. In comparison, the relative percentage composition of VOCs released from the healthy (CK) rice plants was not affected by the CO_2_ level. There are species-specific differences in the response of plant VOCs to the CO_2_ level. Some studies showed that the VOCs’ emission induced by the leaf-chewing herbivores would not be influenced by elevated CO_2_ [71]. However, other studies indicated that the total release of VOCs from *Phaseolus lunatus* significantly increased in response to higher CO_2_ concentration (e.g., Ballhorn et al., 2011) [25]. It was also found that the synthesis of sterols and phytol in the BPH-damaged rice plants was upregulated [72], and the emission of linalool, methyl salicylate and α-zingiberene from the BPH-damaged rice plants was also increased, respectively [73]. Our results also indicated that the host selection of BPH adults was positively correlated with the relative percentage composition of phenols released from rice plants. Meanwhile, the relative percentage composition of 2,6-Diphenylphenol significantly increased under elevated CO_2_. Based on these results, we speculated that more phenolic volatiles were released from rice plants under elevated CO_2_, which would attract more BPH. Therefore, we concluded that the emission of VOCs from host plants would be changed, especially after the damage by herbivorous insects, because of the defensive response of plants to the damage of insect pests. Moreover, the change in the emission of host plant VOCs acted as secondary metabolites, due to a shift in the primary and secondary metabolites under elevated CO_2_, which further repels or attracts herbivorous insects, and finally affects their growth and development by influencing host selection behavior [60,61].

## 5. Conclusions

In conclusion, this study reported that the BPH have stronger host selection abilities for rice plants under elevated CO_2_. A subsequent transcription level study revealed the mechanism of host selection behavior. We explored preliminarily the relationship between the host selection behavior of BPH, and the VOCs of healthy and BPH-damaged rice plants grown under ambient and elevated CO_2_, and found that the phenols play an important role in the selection of rice plants by BPH.

## Figures and Tables

**Table 1:** QRT-PCR primers for the odorant-binding protein genes (*OPB1*, *OBP2* and *OBP3*), chemoreceptor protein genes (*CSP3*, *CSP8* and *CSP10*) and internal reference genes (*Nlβ-Actin* and *Nlug-Actin*) of brown planthopper (BPH), *Nilaparvata lugen*s.

Primer	Sequence (5′-3′)	Description
*NlugOBP1*-F	TTTGGCACAGAAACGATTTGGAG	Odorant-binding protein gene (OBPs)
*NlugOBP1*-R	CATTGGGCACTTGTCTTTGGAG	
*NlugOBP2-*F	CATCAAGAGTGTACCAGAAGGAGAC	
*NlugOBP2-*R	AATCATCAGTTCATACCAGCAAGC	
*NlugOBP3-*F	AAGCCACTGACGAGGATGTAATG	
*NlugOBP3-*R	TTCACACCTTCCAAGTTGAGATTCTG	
*NlugCSP3-*F	TGATTGTGGTCGCGTTTGGA	Chemosensory protein gene (CSPs)
*NlugCSP3-*R	TAGGGCGTCCGGTATTGTTG	
*NlugCSP8-*F	TTTTGTGGCGGTTTTGTGCT	
*NlugCSP8-*R	CCACCCATCAGGCACTTGAA	
*NlugCSP10-*F	AGCTCTGAAAGCCGGACTAC	
*NlugCSP10-*R	ATGAACGCTTTGATGTGGGG	
*Nlβ-Actin-*F	ACTCCGGTGATGGTGTCTCT	Reference genes
*Nlβ-Actin-*R	GTCGGTCAAGTCACGACCA	
*Nlug-Actin-*F	TCAACCCAAAGGCCAACC	
*Nlug-Actin-*R	CACCGGAGTCAAGCACGATA	

**Table 2:** Two-way ANOVAs on the effects of CO_2_ level (ambient CO_2_ versus elevated CO_2_), damaged treatment (no damage (i.e., healthy) rice plants versus BPH-damaged rice plants) and their interaction on the host-selection rate of brown planthopper (BPH) *N. lugens*, and on the percent of eight groups of volatile organic compounds (VOCs) from the healthy (CK) and BPH-damaged rice plants grown under ambient and elevated CO_2_; and one-way ANOVA on the effects of the CO_2_ level on the transcript expression of OBP and CSP genes of *N. lugens* adults fed on rice plants grown under ambient and elevated CO_2_ (*F/p* values).

Indexes		CO_2_ Level (CO_2_)	BPH-Damaged Treatment	CO_2_ × BPH-Damaged Treatment
Host-selection rate (%)		22.022/<0.001 ***	1.011/0.327	4.045/0.058
Odorant-binding proteins (OBPs)	*OBP1*	12.045/0.003 **	/	/
	*OBP2*	16.679/<0.001 ***	/	/
	*OBP3*	0.052/0.823	/	/
Chemosensory proteins (CSPs)	*CSP3*	0.042/0.840	/	/
	*CSP8*	9.462/0.007 **	/	/
	*CSP10*	1.844/0.193	/	/
Volatile organic compounds (VOCs)	Alkane	0.445/0.524	3.783/0.088	2.480/0.154
	Alcohols	0.009/0.926	0.468/0.513	0.607/0.459
	Alkenes	9.195/0.016 *	0.687/0.431	5.160/0.053
	Ketones	0.050/0.829	0.116/0.742	0.243/0.635
	Aldehydes	0.042/0.844	3.992/0.081	0.156/0.703
	Esters	0.002/0.964	9.069/0.017 *	0.249/0.631
	Aromatic hydrocarbons	0.203/0.664	6.917/0.030 *	1.404/0.27
	Phenols	1.914/0.204	27.186/<0.001 ***	7.912/0.023 *

* *p* < 0.05, ** *p* < 0.01, *** *p* < 0.001.

**Table 3:** Two-way ANOVAs on the effects of CO_2_ level (ambient CO_2_ versus elevated CO_2_), damaged treatment and their interaction on the percent of each kind of volatile organic compounds (VOCs) from the healthy (CK) and BPH-damaged rice plants grown under ambient and elevated CO_2_ (*F/p* values).

Volatile Types	VOCs	CO_2_	BPH-Damaged	CO_2_ × BPH-Damaged
Alkane	Undecane	0.011/0.921	4.515/0.066	0.044/0.839
	Tridecane	0.932/0.363	2.022/0.193	0.348/0.572
	Pentadecane	0.170/0.691	0.336/0.578	0.477/0.509
	Hexadecane	0.358/0.566	10.462/0.012 *	0.357/0.567
	Heptadecane	1.670/0.232	0.021/0.888	10.157/0.013 *
	Octadecane	0.203/0.664	0.164/0.696	0.510/0.495
	Icosane	0.491/0.504	2.573/0.147	2.515/0.151
	Tetracosane	0.023/0.884	0.203/0.664	1.505/0.255
	Heptacosane	0.000/0.995	0.011/0.920	0.983/0.350
	5-Ethyl-2,2,3-trimethylheptane	0.302/0.598	0.852/0.383	0.137/0.721
	2,4,6-Trimethyldecane	0.946/0.359	0.000/1.000	0.447/0.523
	4,7-Dimethylundecane	0.037/0.852	2.039/0.191	0.156/0.703
	2,6-Dimethylundecane	0.001/0.975	0.207/0.661	0.067/0.803
	3-Methyltetradecane	0.907/0.369	0.992/0.348	0.920/0.366
	4-Methylpentadecane	1.074/0.330	0.260/0.624	1.929/0.202
	2,6,8-Trimethyldecane	0.003/0.956	0.111/0.748	0.597/0.462
	2,6-Dimethylheptadecane	0.294/0.603	0.016/0.902	1.842/0.212
	4-Methylnonadecane	0.013/0.912	0.040/0.847	0.114/0.744
Alcohols	Linalool	21.416/0.002 **	6.098/0.039 *	9.206/0.016 *
	Trans-2-Undecen-1-ol	0.333/0.580	0.858/0.381	0.008/0.929
	Falcarinol	1.482/0.258	0.686/0.432	0.035/0.855
	2-Hexyl-1-decanol	0.000/0.992	0.136/0.721	0.061/0.811
	Phytol	0.411/0.539	0.612/0.457	1.691/0.230
	Hexacosan-1-ol	0.689/0.431	0.082/0.781	0.418/0.536
	1-Eicosanol	0.531/0.487	0.383/0.553	0.894/0.372
Alkenes	Limonene	12.893/0.007 **	1.758/0.222	7.430/0.026 *
	Myrcene	0.076/0.789	0.537/0.485	1.769/0.220
	1-Docosene	0.171/0.690	0.288/0.606	0.456/0.518
Ketones	2-Dodecanone	0.050/0.829	0.116/0.742	0.243/0.635
Aldehyde	Nonanal	0.011/0.918	10.012/0.013 *	0.105/0.754
	Decanal	0.164/0.696	4.718/0.062	0.056/0.819
	(Z)-9-Tetradecenal	0.045/0.838	0.421/0.535	0.836/0.387
Esters	Methyl cis-9,10-epoxystearate	0.003/0.955	9.180/0.016 *	0.275/0.614
Aromatic hydrocarbon	1-Methyldecalin	2.165/0.179	3.262/0.109	4.603/0.064
	2-Methyltetralin	0.298/0.600	3.136/0.115	0.031/0.865
Phenols	2,6-Diphenylphenol	1.914/0.204	27.186/<0.001 ***	7.912/0.023 *

BPH-Brown planthopper, *N. lugens*; * *p* < 0.05, ** *p* < 0.01, *** *p* < 0.001.

**Table 4:** Composition and percentage (%) of the volatile organic compounds (VOCs) from the healthy (CK) and BPH-damaged rice plants grown under ambient and elevated CO_2._

Volatile Types	VOCs	Healthy (CK) Rice Plants		BPH-Damaged Rice Plants	
		Ambient CO_2_	Elevated CO_2_	Ambient CO_2_	Elevated CO_2_
Alkane	Undecane	16.61 ± 1.12	16.43 ± 0.10b	17.72 ± 0.18	17.78 ± 0.21a
	Tridecane	21.51 ± 0.69	21.70 ± 0.48	21.94 ± 0.64	22.76 ± 0.04
	Pentadecane	6.83 ± 0.09	6.68 ± 0.03	6.81 ± 0.19	6.85 ± 0.16
	Hexadecane	3.78 ± 0.10	3.78 ± 0.05b	3.95 ± 0.04	4.03 ± 0.04a
	Heptadecane	8.47 ± 0.03	9.26 ± 0.34	9.06 ± 0.08A	8.72 ± 0.02B
	Octadecane	2.47 ± 0.17	2.36 ± 0.01	2.37 ± 0.05	2.39 ± 0.01
	Icosane	10.58 ± 0.41	9.84 ± 0.46	9.55 ± 0.13	9.84 ± 0.14
	Tetracosane	4.84 ± 0.31	4.47 ± 0.23	4.40 ± 0.28	4.68 ± 0.23
	Heptacosane	3.61 ± 0.29	3.37 ± 0.31	3.40 ± 0.16	3.64 ± 0.18
	5-Ethyl-2,2,3-trimethylheptane	0.19 ± 0.01	0.20 ± 0.02	0.21 ± 0.01	0.21 ± 0.02
	2,4,6-Trimethyldecane	0.21 ± 0.01	0.21 ± 0.01	0.22 ± 0.00	0.21 ± 0.00
	4,7-Dimethylundecane	0.94 ± 0.11	0.96 ± 0.02	1.07 ± 0.03	1.03 ± 0.09
	2,6-Dimethylundecane	0.53 ± 0.03	0.52 ± 0.01	0.53 ± 0.02	0.54 ± 0.01
	3-Methyltetradecane	0.63 ± 0.00	1.14 ± 0.53	0.62 ± 0.02	0.62 ± 0.02
	4-Methylpentadecane	0.83 ± 0.05	0.75 ± 0.01	0.77 ± 0.04	0.78 ± 0.02
	2,6,8-Trimethyldecane	2.26 ± 0.21	2.19 ± 0.03	2.14 ± 0.06	2.23 ± 0.04
	2,6-Dimethylheptadecane	3.19 ± 0.23	2.94 ± 0.08	3.03 ± 0.08	3.14 ± 0.08
	4-Methylnonadecane	0.42 ± 0.05	0.41 ± 0.06	0.39 ± 0.03	0.41 ± 0.03
Alcohols	Linalool	0.19 ± 0.003b	0.17 ± 0.01	0.23 ± 0.00Aa	0.17 ± 0.01B
	Trans-2-Undecen-1-ol	0.29 ± 0.01	0.29 ± 0.01	0.30 ± 0.01	0.30 ± 0.01
	Falcarinol	0.59 ± 0.07	0.52 ± 0.02	0.65 ± 0.08	0.56 ± 0.06
	2-Hexyl-1-decanol	1.28 ± 0.19	1.26 ± 0.06	1.22 ± 0.02	1.25 ± 0.02
	Phytol	0.37 ± 0.07b	0.87 ± 0.22	0.91 ± 0.10a	0.74 ± 0.45
	Hexacosan-1-ol	1.24 ± 0.06	1.16 ± 0.09	1.22 ± 0.04	1.21 ± 0.02
	1-Eicosanol	0.83 ± 0.06	0.76 ± 0.05	0.76 ± 0.04	0.77 ± 0.01
Alkenes	Limonene	0.78 ± 0.19	0.67 ± 0.15	1.30 ± 0.08A	0.49 ± 0.02B
	Myrcene	0.16 ± 0.03	0.18 ± 0.03	0.17 ± 0.03	0.13 ± 0.02
	1-Docosene	0.67 ± 0.05	0.63 ± 0.04	0.63 ± 0.02	0.64 ± 0.03
Ketones	2-Dodecanone	0.12 ± 0.03	0.13 ± 0.02	0.13 ± 0.02	0.11 ± 0.03
Aldehyde	Nonanal	0.44 ± 0.03	0.42 ± 0.00b	0.56 ± 0.08	0.57 ± 0.03a
	Decanal	0.18 ± 0.02b	0.20 ± 0.06	0.26 ± 0.02a	0.27 ± 0.02
	(Z)-9-Tetradecenal	0.72 ± 0.07	0.77 ± 0.02	0.73 ± 0.03	0.70 ± 0.06
Esters	Methyl cis-9,10-epoxystearate	3.05 ± 0.88	3.48 ± 1.04	1.21 ± 0.55	0.87 ± 0.04
Aromatic hydrocarbon	1-Methyldecalin	0.29 ± 0.01b	0.33 ± 0.01	0.33 ± 0.01a	0.32 ± 0.01
	2-Methyltetralin	0.34 ± 0.02	0.33 ± 0.02	0.36 ± 0.01	0.36 ± 0.00
Phenols	2,6-Diphenylphenol	0.55 ± 0.02b	0.61 ± 0.02b	0.85 ± 0.07a	0.69 ± 0.01a

BPH-Brown planthopper, *N. lugens*; Different uppercase and lowercase letters indicate significant difference between ambient and elevated CO_2_, and between CK and BPH-damaged by the independent sample *t* test at *p* < 0.05.

## Data Availability

The data obtained in this study has been presented “as is” on at least one of the figures or tables embedded in the manuscript.

## Supplementary Materials

The following supporting information can be downloaded at: https://doi.org/10.6084/m9.figshare.20017538. All original data can be found in it.
